# *Gymnema inodorum* (Lour.) Decne. Extract Alleviates Oxidative Stress and Inflammatory Mediators Produced by RAW264.7 Macrophages

**DOI:** 10.1155/2021/8658314

**Published:** 2021-02-04

**Authors:** Benjawan Dunkhunthod, Chutima Talabnin, Mark Murphy, Kanjana Thumanu, Patcharawan Sittisart, Griangsak Eumkeb

**Affiliations:** ^1^School of Preclinical Sciences, Institute of Science, Suranaree University of Technology, Nakhon Ratchasima 30000, Thailand; ^2^School of Chemistry, Institute of Science, Suranaree University of Technology, Nakhon Ratchasima 30000, Thailand; ^3^School of Biomolecular Science, Liverpool John Moores University, Liverpool L3 3AF, UK; ^4^Synchrotron Light Research Institute (Public Organization), Nakhon Ratchasima 30000, Thailand; ^5^Division of Environmental Science, Faculty of Liberal Arts and Science, Sisaket Rajabhat University, Sisaket 33000, Thailand

## Abstract

*Gymnema inodorum* (Lour.) Decne. (*G. inodorum*) is widely used in Northern Thai cuisine as local vegetables and commercial herb tea products. In the present study, *G. inodorum* extract (GIE) was evaluated for its antioxidant and anti-inflammatory effects in LPS plus IFN-*γ*-induced RAW264.7 cells. Major compounds in GIE were evaluated using GC-MS and found 16 volatile compounds presenting in the extract. GIE exhibited antioxidant activity by scavenging the intracellular reactive oxygen species (ROS) production and increasing superoxide dismutase 2 (SOD2) mRNA expression in LPS plus IFN-*γ*-induced RAW264.7 cells. GIE showed anti-inflammatory activity through suppressing nitric oxide (NO), proinflammatory cytokine production interleukin 6 (IL-6) and also downregulation of the expression of cyclooxygenase-2 (COX-2), inducible nitric oxide synthase (iNOS), and IL-6 mRNA levels in LPS plus IFN-*γ*-induced RAW264.7 cells. Mechanism studies showed that GIE suppressed the NF-*κ*B p65 nuclear translocation and slightly decreased the phosphorylation of NF-*κ*B p65 (p-NF-*κ*B p65) protein. Our studies applied the synchrotron radiation-based FTIR microspectroscopy (SR-FTIR), supported by multivariate analysis, to identify the FTIR spectral changes based on macromolecule alterations occurring in RAW264.7 cells. SR-FTIR results demonstrated that the presence of LPS plus IFN-*γ* in RAW264.7 cells associated with the increase of amide I/amide II ratio (contributing to the alteration of secondary protein structure) and lipid content, whereas glycogen and other carbohydrate content were decreased. These findings lead us to believe that GIE may prevent oxidative damage by scavenging intracellular ROS production and activating the antioxidant gene, SOD2, expression. Therefore, it is possible that the antioxidant properties of GIE could modulate the inflammation process by regulating the ROS levels, which lead to the suppression of proinflammatory cytokines and genes. Therefore, GIE could be developed into a novel antioxidant and anti-inflammatory agent to treat and prevent diseases related to oxidative stress and inflammation.

## 1. Introduction

Inflammation is the immune system's response to harmful stimuli, such as infection and tissue injury [1]. Macrophages are a diverse group of white blood cells known for eliminating pathogens through phagocytosis. Macrophages play a central role in promoting inflammatory lesions, which cause pathological tissue damage in various inflammatory diseases [2]. It is well known that interferon-gamma (IFN-*γ*) or lipopolysaccharide (LPS) is sufficient to induce classically activated macrophages [3]. The nuclear factor kappa light chain enhancer of activated B cells (NF-*κ*B) is an important transcription factor playing crucial roles in the inflammatory response [4]. In response to inflammatory stimuli, the nuclear localization signal of cytosolic NF-*κ*B into the nucleus binds to a consensus sequence in the promoters of target genes including proinflammatory cytokines, chemokines, adhesion proteins, and inducible enzymes (COX-2 and iNOS) [5]. Due to the response with the existence of cellular stimuli, activated macrophages produce high levels of proinflammatory cytokines, including IL-6, interleukin 1*β* (IL-1*β*), TNF-*α*, and prostaglandin (PGE_2_) [6]. These cytokines are involved in the process of pathological pain. During inflammation, free radical molecules such as NO and reactive oxygen intermediates (ROI) are also generated by inflammatory cells. Free radicals are involved in an imbalance of the redox system and damaging cells and tissues [7]. The link between oxidative stress and inflammation has extensively been demonstrated that the mechanism by which continued oxidative stress can lead to chronic inflammation. This mechanism leads to various inflammatory diseases such as diabetes, cardiovascular diseases, cancer, degenerative diseases, ischemia, and anemia [8]. Currently, the available drug in treating inflammatory disorders is often not successful, lacking availability, high cost, and causes of undesirable side effects [9, 10]. Based on these, the necessity for exploring a better anti-inflammatory therapeutic agent is always in need.

Herbs' use as traditional medicine has a long history in treating various diseases, including inflammatory diseases. It is currently well known that many bioactive compounds derived from natural products play an essential role in a wide range of therapeutic effects [11]. The World Health Organization (WHO) recognizes the vital role of traditional medicines and continues to support the integration of conventional medicine into each country's health system [12].

*G. inodorum* is one of the local Thai vegetables belonging to the family Asclepiadaceae. It is found ubiquitously in Southeastern Asia, including Thailand, especially in the northern region. In Thailand, its local name is “Phak chin da” or “Phak chiang da.” It has been known to have therapeutic effects in curing certain diseases, including diabetes mellitus, rheumatic arthritis, and gout. The literature survey reported that the leaves of *G. inodorum* had many phytochemical compounds such as phenolics, flavonoids, terpenoids, and glycoside [13, 14]. Moreover, its antioxidant, antiadipogenesis, antidiabetic, and hypoglycemic effects were also reported [14–16]. Therefore, the phytochemical constituents presented in *G. inodorum* may contribute to its biological activity. Numerous studies exhibited that the phytochemical compounds, especially flavonoids and phenolic content, had contributed to the redox-modulating properties of natural compounds, which had also been shown to modulate the inflammatory response virtually [15–17]. Along this line, it is possible that *G. inodorum* may exert a beneficial effect on alleviating intracellular ROS and inflammation. However, to the best of our knowledge, this plant's antioxidant and anti-inflammatory activities have not earlier been reported, especially for the research study in cell-based assays.

Fourier-transform infrared spectroscopy (FTIR) is an analytical technique widely applied for studying the vibrational fingerprint for organic compounds [17]. This technique was previously applied to study biomedical research to investigate biomolecule profiles (lipid, protein, nucleic acids, and carbohydrate) in various biological samples without the need for probe molecules [18–20]. Synchrotron light is exceptionally bright. When a synchrotron light (SR) source, FTIR spectroscopy, and microscopy are combined together, it is called “Synchrotron radiation-based FTIR microspectroscopy (SR-FTIR).” This technique takes advantage of synchrotron light brightness, making it possible to record high-quality spectra and explore the biochemical changes within biological samples' microstructures without destruction's inherent structures of its [21].

The present study is aimed at investigating the anti-inflammatory and the antioxidant effect of GIE on LPS plus IFN-*γ*-induced RAW264.7 cells. The SR-FTIR was applied to detect biomolecule changes in RAW264.7 cells induced by LPS plus IFN-*γ* and their response to GIE treatment.

## 2. Materials and Methods

### 2.1. Preparation of GIE

*G. inodorum* (dried leaves) was purchased from commercial products (Chiangda organic company garden, Chiangmai, Thailand). The voucher specimens were deposited at the botanical garden of Suranaree University of Technology (SUT) Herbarium and authenticated by Dr. Santi Wattatana, a lecturer and a plant biologist at the Institute of Science, SUT, Thailand. The plant extractions were conducted following the method of Tiamyom et al. [14]. Briefly, the dried powder of *G. inodorum* was soaked in 95% ethanol with a ratio of 1 : 3 (g : mL) at room temperature for 7 days. The pooled extract was filtered through Whatman No. 1 filter paper. The ethanolic extract was concentrated using a vacuum rotary evaporator at 50°C and lyophilized to obtain the powder of GIE. The crude extract was stored at -20°C till use in subsequent experiments. GIE was dissolved in dimethyl sulfoxide (DMSO) and diluted to 0.1% (*v*/*v*) in the cell culture medium.

### 2.2. Identification of Phytochemical Constituents of GIE Gas Chromatography-Mass Spectrometry (GC-MS) Analysis

GC-MS analysis of GIE was performed using a Bruker 450-GC/Bruker 320-MS equipped with Rtx-5MS fused silica capillary column (30 m length × 250 *μ*m diameter × 0.25 *μ*m film thickness). For GC-MS detection, an electron ionization system was operated in the electron impact mode with ionization energy of 70 eV. The injector temperature was maintained at 250°C, and the ion-source temperature was 200°C. The oven temperature was programmed from 110°C (2 min), with an increase of 10°C/min to 200°C (3 min), then 5°C/min to 280°C, ending with a 20 min isothermal at 280°C. The MS scan range was 45-500 atomic mass units (amu), and helium was used as the carrier gas with a flow rate of 1.0 mL/min. The chemical components were identified by matching their mass spectra with those recorded in the NIST mass spectral library 2008.

### 2.3. Ferric Reducing/Antioxidant Power (FRAP) Assay

The ferric reducing capacity of GIE was determined by using the colorimetric method as described by Rupasinghe et al. [22]. Briefly, 20 *μ*L of each of the samples and 180 *μ*L of FRAP reagent (300 mM acetate buffer (pH 3.6), 10 mM 2, 4, 6-tripyridyl-s-triazine (TPTZ), and 20 mM FeCl_3_•6H_2_O solution) were mixed in a 96-well plate for 6 min. The absorbance was recorded at 595 nm using a microplate reader (Bio-Rad Laboratories, Inc., Hercules, CA, USA). The different concentrations of Trolox (Cat. No. 238813, Sigma-Aldrich, St. Louis, USA) and vitamin C (Cat. No. 95210, Fluka Chemie GmbH, Buchs, Switzerland) were used to develop the standard calibration curve. FRAP values were expressed as a milligram of Trolox equivalent antioxidant capacity (TREA) or vitamin C equivalent antioxidant capacity (VCEA) per gram of dry extract.

### 2.4. 2,2-Diphenyl-1-Picryl-Hydrazyl (DPPH) Radical-Scavenging Activity Assay

The ability of GIE to scavenge the DPPH radical was estimated according to the method of Yang et al. [23]. Briefly, an aliquot of 100 *μ*L of the sample at different concentrations was added to 100 *μ*L of 0.2 mM DPPH solution (Cat. No. D9132, Sigma-Aldrich, St. Louis, USA) in a 96-well plate. The reaction mixture was kept in the dark for 15 min, and the absorbance was measured at 517 nm using a microplate reader (Bio-Rad Laboratories, Inc., Hercules, CA, USA). The positive standards (Trolox and vitamin C) were prepared using the same procedure. A lesser absorbance rate demonstrated higher radical scavenging activity. The percentage inhibition of free radical was calculated using the following equation: %Inhibition = [(*A*_control_ − *A*_sample_)/*A*_control_] × 100. The sample concentration providing 50% inhibition (IC_50_) was determined from a dose-response curve using linear regression analysis.

### 2.5. Cell Culture

The murine macrophage cell line RAW264.7 (CLS Cell Lines Service GmbH., Eppelheim, Germany) was cultured in RPMI-1640 medium (Cat. No. 1IVG1-31800022) supplemented with 10% heat-inactivated fetal bovine serum (FBS, Cat. No. 1IVG7-10270-106) and 100 U/mL penicillin-streptomycin (Cat. No. 1IVG7-15140-122) (GIBCO, Grand Island, NY, USA). Cells were maintained at 37°C in 95% humidified with 5% of the CO_2_ atmosphere.

### 2.6. Measurement of Cell Proliferation by the 3-(4,5-Dimethylthiazol-2-yl)-2,5-Diphenyl-Tetrazolium Bromide (MTT) Assay

The effect of GIE on cell viability was evaluated by using a tetrazolium dye colorimetric assay as described by Tiamyom et al. [14] and Dunkhunthod et al. [24]. Briefly, RAW264.7 cells were seeded in a 96-well plate (2 × 10^4^ cells/well) and cultured for 24 h. Cells were treated with different concentrations of GIE for 24 h. Following treatment, the culture medium was removed, the MTT solution (0.5 mg/mL) (Cat. No. M6494, Invitrogen, Carlsbad, CA, USA) was added and further incubated at 37°C for 4 h. Subsequently, 150 *μ*L DMSO was added to dissolve formazan crystal formed by viable cells, and absorbance was measured at 540 nm with a microplate spectrophotometer (Bio-Rad Laboratories, Inc., Hercules, CA, USA).

### 2.7. Detection of Intracellular ROS by 2′,7′-Dichlorofluorescein-Diacetate (DCFH-DA) Assay

Relative changes of intracellular ROS in RAW264.7 cells were monitored using the fluorescent probe DCFH-DA (Cat. No. D6883, Sigma-Aldrich, St. Louis, USA) as previously described by Sittisart and Chitsomboon [25]. Briefly, RAW264.7 cells were seeded onto a 96-well black plate at 2.0 × 10^4^ cells/well and cultured for 24 h. After removing the medium, the cells were pretreated with GIE at the concentration of 50, 100, 200, or 300 *μ*g/mL or a selective ROS scavenger, N-acetyl-cysteine 3 mM (NAC, Cat. No. A9165, Sigma-Aldrich, St. Louis, USA) for 3 h prior to exposing them to 1 *μ*g/mL lipopolysaccharide (LPS) (LPS from *Escherichia coli* O111:B4, Cat. No. L2630, Sigma-Aldrich, St. Louis, USA) plus 10 ng/mL IFN-*γ* (Cat. No. 200-16, Shenandoah Biotechnology Inc., Warwick, PA, USA) for 24 h. After removing the medium and washing the cells with PBS twice, 20 *μ*M DCFH-DA in Hank's Balanced Salt Solution (HBSS, Cat. No. TFS-CB-14175095, GIBCO, Grand Island, NY, USA) was then added, and the cells were incubated for 30 min at 37°C. The cells were washed twice with phosphate-buffered saline (PBS), and fluorescence intensity was measured using a Gemini EM fluorescence microplate reader (Molecular Devices, Sunnyvale, CA, USA) with an excitation wavelength of 485 nm and an emission wavelength of 535 nm. Data were expressed as the percentage of 2′-7′-dichlorofluorescein (DCF) fluorescence intensity that was calculated according to the following formula: DCF fluorescence intensity (%) = (DCF fluorescence intensity_test group_/DCF fluorescence intensity_control group_) × 100.

### 2.8. Cell Treatment

The RAW 264.7 cells (6 × 10^5^ cells/well) were seeded in a 6-well plate and cultured for 24 h. The cells were pretreated with GIE at the concentration of 50, 100, 200, or 300 *μ*g/mL or 1 *μ*M dexamethasone (DEX, Cat. No. API-04, G Bioscience, St. Louis, MO, USA) or 300 *μ*g/mL of vitamin E (Vit.E, Cat. No. 95240, Fluka Chemie GmbH, Buchs, Switzerland) for 3 h prior to exposing them to 1 *μ*g/mL LPS plus 10 ng/mL IFN-*γ* for 24 h.

### 2.9. Observation of Morphological Changes by Hematoxylin Staining

The phenotype feature of RAW264.7 cells was observed by staining with hematoxylin solution described by Dunkhunthod et al. [24] and visualized under the inverted fluorescence microscope (Olympus Corporation, Shinjuku, TYO, Japan).

### 2.10. Determination of NO by Griess Reagents

After treatment, the culture supernatants were collected for analysis of NO using Griess reagents. The accumulation of nitrite in culture supernatants as an indicator of NO production by RAW264.7 cells was evaluated using Griess reagent (Cat. No. 109023, Merck KGaA, Darmstadt, Germany) as described by Sittisart and Chitsomboon [25]. The amount of nitrite in the samples was calculated using the linear sodium nitrite calibration curves at a concentration range of 2.5-100 *μ*M.

### 2.11. Determination of Proinflammatory Cytokines (IL-6 and TNF-*α*) and Anti-inflammatory Cytokines (IL-10) by ELISA

After treatment, the supernatants containing antigens were collected. The levels of IL-6, TNF-*α*, and IL-10 were quantified by DuoSet® ELISA Kits (IL-6; Cat. No. DY406-05, TNF-*α*; Cat. No. DY410-05, and IL-10; Cat. No. DY417-05, R & D systems Inc., Minneapolis, MN, USA) according to the manufacturer's instructions. The *optical density* of each well was measured at 450 nm with a microplate reader (Bio-Rad Laboratories, Inc., Hercules, CA, USA). The number of cytokines in the samples was calculated using standard cytokine linear calibration curves at indicated concentration ranges.

### 2.12. Detection of mRNA Expression by Quantitative Real-Time Polymerase Chain Reaction (qRT-PCR)

To determine the level of mRNA expression of inflammatory genes (iNOS and COX-2) and antiantioxidant genes, including SOD2, glutathione S-transferase pi 1 (GSTP1), NAD(P)H quinone dehydrogenase 1 (NQO1), cysteine ligase catalytic subunit (GCLC), and glutamate-cysteine ligase regulatory subunit (GCLM) in RAW264.7 macrophage cells after treating with GIE. Total RNA was isolated using TRIzol reagent (Cat. No. 15-596-026, Invitrogen, Carlsbad, CA, USA), and 2 *μ*g of total RNA was reverse transcribed to single-stranded cDNA using the SuperScript VILO cDNA Synthesis Kit (Cat. No. 11754-050, Invitrogen™, California, USA) at 42°C for 1 h. qPCR was performed in a LightCycler® 480 Real-Time PCR System (Roche Diagnostics, Mannheim, Germany) using SYBR green master mix. The PCR was performed in a final volume of 20 *μ*L containing 1 *μ*L of primer mixture (10 *μ*M), 10 *μ*L of 2X SYBR Green Master Mix (Cat. No. 04707516001, Roche Diagnostics, Mannheim, Germany), 5 *μ*L of cDNA template (5 *μ*g), and 4 *μ*L of nuclease-free distilled water. Real-time PCR cycles included initial denaturation at 95°C for 5 min, 95°C for 10 s, annealing at 60°C for 20 s, and extension at 72°C for 30 s through 40 cycles. The specificity of each of the PCR products was confirmed by melting curve analysis. The fold change in mRNA expression was calculated by comparing the GAPDH normalized threshold cycle numbers (Ct) in the untreated- and GIE treated-LPS plus IFN-*γ*-induced cells compared to the uninduced cells using the 2^−ΔΔCt^ method. Triplicate wells were run for each experiment, and two independent experiments were performed. The primer sequences designed for qRT-PCR analysis are listed in Table 1.

### 2.13. Western Blotting Analysis

The expression of NF-*κ*B p65 and p-NF-*κ*B p65 in RAW264.7 macrophage cells after treating with GIE for 24 h was examined. After incubation, the cells were washed three times with PBS and placed in 150 *μ*L of ice-cold lysis buffer (1 mL RIPA buffer supplemented with 2 mM phenylmethylsulfonyl fluoride (PMSF), 2 *μ*M leupeptin, and 1 *μ*M E-64) for 20 min. The disrupted cells were then transferred to microcentrifuge tubes and centrifuged at 14,000 g at 4°C for 30 min. The supernatant was collected, and the protein concentration of cell lysate was estimated by the Lowry method [26]. Thirty micrograms of cellular proteins were separated by sodium dodecyl sulfate-polyacrylamide gel electrophoresis (SDS-PAGE) using 10% polyacrylamide gels (125 volts, 120 min). The proteins in the gel were transferred onto a nitrocellulose membrane (Amersham, Pittsburgh, PA, USA) at 80 volts for 1 h. The membrane was blocked with 5% bovine serum albumin (BSA) in 0.1% Tween 20 in a PBS buffer (0.1% PBST) at room temperature for 1 h. The membranes were then incubated with a 1 : 1,000 dilution of the mouse monoclonal anti-NF-*κ*B p65 antibody (F-6, Cat. No. sc-8008), anti-p-NF-*κ*B p65 (27.Ser 536, Cat. No. sc136548), and *α*-tubulin (B-7, Cat. No. sc-5286) (Santa Cruz Biotechnology, Inc., Dallas, TX, USA) at 4°C overnight. After extensive washing with 0.1% PBST, the membranes were incubated with a 1 : 5,000 dilution of the secondary antibody mouse IgG*κ* light chain binding protein conjugated to horseradish peroxidase (m-IgG*κ* BP-HRP, Cat. No. sc-516102, Santa Cruz Biotechnology, Inc., Dallas, TX, USA) at room temperature for 1 h. The membranes were washed three times for 5 min each time, with 0.1% PBST. The membranes were incubated for 3 min in SuperSignal™ West Pico Chemiluminescent Substrate (Cat. No. 34079, Thermo Scientific, Waltham, MA, USA) and exposed to film. The relative expression of NF-*κ*B p65 and p-NF-*κ*B p65 proteins was quantified densitometrical using the software image J. The *α*-tubulin was used as a housekeeping protein.

### 2.14. Immunofluorescence Staining

As a marker of NF-*κ*B activation, the NF-*κ*B p65 subunit's nuclear translocation was visualized in RAW264.7 cells by immunofluorescence microscopy. Cells were seeded at a density of 6 × 10^4^ cells/well in an 8-well cell culture slide. After 24 h of incubation, cells were pretreated with 300 *μ*g/mL GIE or 1 *μ*M of DEX for 3 h. Then, cells were coincubated with 1 *μ*g/mL LPS+10 ng/mL IFN-*γ* for another 24 h. After incubation, cells were fixed with 4% paraformaldehyde for 15 min and then permeabilized with 0.1%Triton-X100 for 10 min at room temperature. After washing with PBS, the samples were blocked in 0.1% PBST containing 4% BSA and then incubated overnight at 4°C with the mouse monoclonal anti-NF-*κ*B p65 antibody (F-6, Cat. No. sc-8008, Santa Cruz Biotechnology, Inc., Dallas, TX, USA) diluted 1 : 200 in 0.1% PBST containing 1% BSA. After washing with 0.1% PBST, each reaction was followed by incubation for 1 h with anti-mouse conjugated to Alexa 488-conjugated goat anti-mouse IgG (Cat. No. ab150113, Abcam, Cambridge, UK) diluted 1 : 250 in 0.1% PBST containing 1% BSA. After washing with 0.1% PBST, the cells were incubated with 10 mg/mL Hoechst 33258 diluted 1 : 2000 in 0.1% PBST (Cat. No. H3569, Invitrogen, Waltham, MA, USA) for 10 min at room temperature and then washed with 0.1% PBST. Slides were mounted with Bio Mount HM mounting medium (Cat. No. 05-BMHM100, Bio-Optica Milano S.p.a., Milano, Italy). Images of the fixed RAW264.7 cells were taken with confocal microscopy (Nikon, Melville, NY, USA).

### 2.15. Detection of Biomolecule Changing by SR-FTIR Microspectroscopy

FTIR experiments were conducted using a spectroscopy facility at the Synchrotron Light Research Institute (Public Organization), Nakhon Ratchasima, Thailand. Sample preparation was performed, as previously described by Dunkhunthod et al. [24]. FTIR spectra were acquired in transmission mode with a Vertex 70 FTIR spectrometer coupled with an IR microscope Hyperion 2000 (Bruker Optics, Ettlingen, Germany), using synchrotron radiation as an IR source. The microscope was equipped with a 64 × 64 element MCT, FPA detector, which allowed simultaneous spectral data acquisition with a 36× objective. The measurements were performed using an aperture size of 10 *μ*m × 10 *μ*m with a spectral resolution of 4 cm^−1^, with 64 scans coadded. FTIR spectrum was recorded within a spectral range of 4000-600 cm^−1^ using OPUS software (Bruker Optics Ltd., Ettlingen, Germany).

Unsupervised explorative multivariate data analysis by Principal Component Analysis (PCA) was conducted using variables within a spectral range of 3,000-2,800 cm^−1^ and 1,800-950 cm^−1^. Data manipulations were processed following the method of Tiamyom et al. [14] with slight modification. Briefly, the data were preprocessed by taking the second derivative using the Savitzky-Golay algorithm (with 15 points of smoothing), followed by normalization with extended multiplicative signal correction (EMSC) (Unscrambler® X software version 10.5, CAMO Software AS, Oslo, Norway). The outcome of the analysis could be presented as 2D score plots and loading plots. The integrated peak areas of the second derivative FTIR spectra were calculated using OPUS 7.2 software (Bruker Optics, Ettlingen, Germany) in the lipid regions (3000-2800 cm^−1^), nucleic acids regions (1257-1204 cm^−1^ and 1125-1074 cm^−1^), and glycogen and other carbohydrates regions (1181-1164 cm^−1^ and 1063-1032 cm^−1^). The band area ratio of amide I to amide II was obtained by calculating the ratio of the amide I (1670-1627 cm^−1^) area to the area under the amide II (1558-1505 cm^−1^) regions.

### 2.16. Statistical Analysis

All data are expressed as the means ± S.D. from at least three independent experiments. The statistical significances (Statistical Package for the Social Sciences, version 19) were determined by performing a one-way analysis of variance (ANOVA). Tukey's test was used as a *post hoc* test. *p* < 0.05 was considered as statistically significant difference, which was indicated by the different superscript letters.

## 3. Results and Discussion

### 3.1. GC-MS Analysis of Volatile Oil Constituents of GIE

In this study, the phytochemical constituents of GIE were analyzed. GC-MS analysis in GIE reported about 16 compounds (as shown in Table 2). The major prevailing compounds were linolenic acid (24.91%), n-Hexadecanoic acid (Palmitic acid) (16.98%), and Methylparaben (11.58%). Besides, it also presented the other chemical compounds, which are known to exhibit important pharmacological activity, in particular, anti-inflammatory and antioxidant activities such as phytol [27, 28], squalene [29], *γ*-tocopherol, dl-*α*-tocopherol [30, 31], and stigmasterol [32, 33].

Phytol, diterpene alcohol, has been reported to have remarkable anti-inflammatory activity by reducing carrageenan-induced paw edema and inhibiting the recruitment of total leukocytes and neutrophils, a decrease in IL-1*β* and TNF-*α* levels and oxidative stress [27]. Jeong [28] also reported that phytol suppressed H_2_O_2_-induced inflammation, as indicated by the reduced expression of the mRNA levels of TNF-*α*, IL-6, IL-8, and COX-2. Squalene, a natural lipid belonging to the terpenoid family, significantly inhibited the secretion of proinflammatory cytokines (TNF-*α*, IL-1*β*, IL-6, and IFN-*γ*), proinflammatory enzymes (iNOS, COX-2, and myeloperoxidase (MPO)), and enhanced expression levels of anti-inflammatory enzymes (heme oxygenase-1 (HO-1)) and transcription factors (Nrf2 and PPAR*γ*) in overactivation of neutrophils, monocytes, and macrophages [29]. *γ*-Tocopherol and dl-*α*-tocopherol exhibited anti-inflammatory activity *in vitro* and *in vivo*, whereas the combination of *γ*- and *α*-tocopherols seems to be more potent than supplementation with *α*-tocopherols alone [30]. These results provide evidence that GIE is a source of antioxidants and anti-inflammatory agents, which could have beneficial effects on treating inflammation-related diseases. However, further studies are needed to clarify pharmacological properties.

### 3.2. Antioxidant Capacity of GIE

Antioxidants can act via several pathways. Therefore, to investigate the antioxidant activity of the GIE, we estimated their reducing antioxidant power and free radical scavenging by using the FRAP and DPPH radical scavenging assays, respectively. Trolox and vitamin C were used as standard antioxidant compounds. As shown in Table 3, GIE exhibited antioxidant activity due to its ability to reduce ferric ion (Fe^3+^) to ferrous ion (Fe^2+^) by 24.00 ± 0.69 *μ*g VCEA/mg of dry extract and 28.06 ± 0.78 *μ*g TREA/mg of dry extract. GIE at a concentration of 406.59 ± 0.11 *μ*g/mL displayed the ability to scavenge DPPH radical at 50% (IC_50_). In contrast, the positive antioxidant controls, vitamin C, and Trolox exhibited the IC_50_ values of approximately 44.57 ± 0.59 *μ*g/mL and 67.19 ± 4.82 *μ*g/mL, respectively.

Based on GC-MS analysis (Table 2), GIE contained many bioactive compounds such as phytol, squalene, *γ*-tocopherol, dl-*α*-tocopherol, and stigmasterol, which were reported to have antioxidant activity. Previous studies demonstrated that the phytochemical screening of GIE indicated the presence of phenolic, flavonoids, terpenoids, and glycoside [14, 15]. Numerous studies have shown that plant products' antioxidant capacity is related to their phenolic and flavonoid contents [34–36]. The synergistic effect of some substances present in GIE had been reported the antioxidant activity. Previous studies showed that the combination of squalene and *α*-tocopherol displayed a synergistic effect by reducing the rate of oxidation in a crocin bleaching assay where squalene might act as a competitive compound to *α*-tocopherol [37]. The sunflower seed oil containing total polyphenols and *α*-tocopherol had a positive *K*_a_/*K*_c_ ratio of rate constants for antioxidant and crocin value antioxidant activity. It is possible that the synergistic effect occurs between *α*-tocopherol and total polyphenols [38]. Besides, the presence of *α*-, *β*-, *γ*-tocopherol in sunflower seed oil may contribute to the oil resistance to oxidation [38, 39]. Therefore, the presence of squalene, *α*-tocopherol, *γ*-tocopherol, and phenolic in GIE may provide synergistic effects on antioxidant activity. However, it is also essential to further confirm whether the antioxidant activity of the combination of *α*-tocopherol plus squalene or other volatile compounds in GIE is synergistic. Moreover, the synergistic action of such compounds from plants had been calculated by the combination index (CI) [40–42].

### 3.3. Cytotoxic Effects of GIE on RAW264.7 Cells

**Figure 1:**
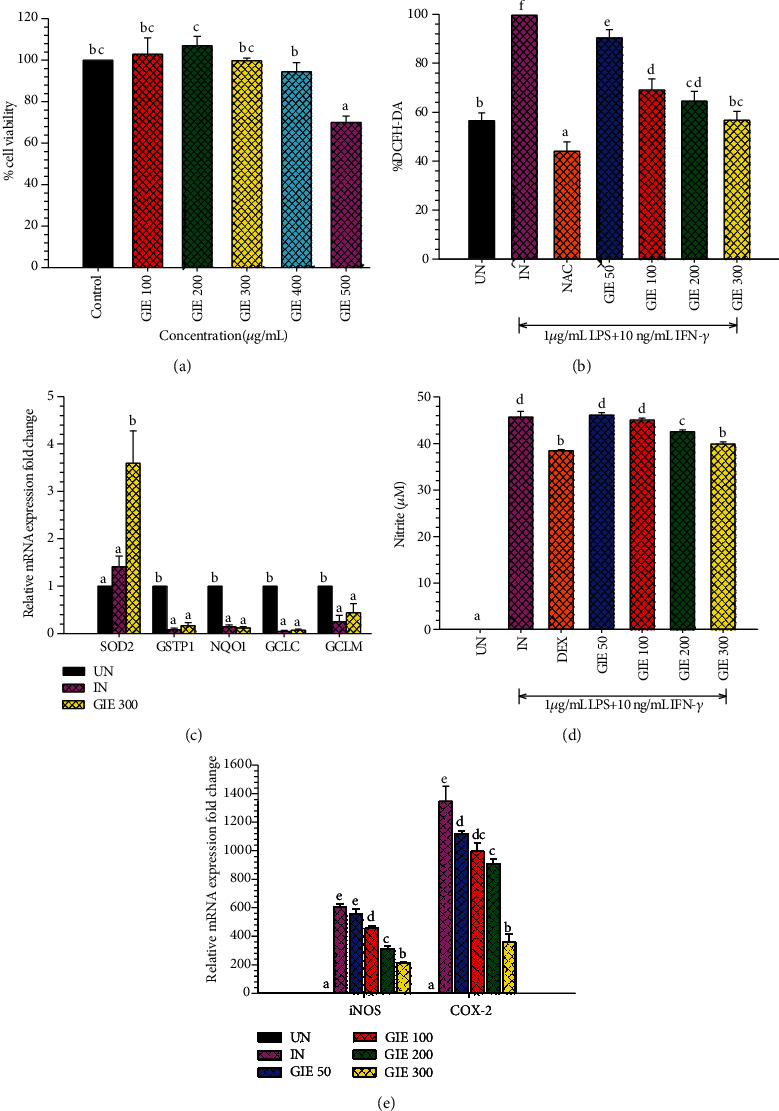
(a) Cytotoxic effects of GIE on RAW264.7 cells. Cells were treated with different concentrations of GIE (100-500 *μ*g/mL) for 24 h. MTT assay was used to determine cell viability. Values are expressed as a percentage of the control. (b) GIE attenuated the intracellular ROS production in LPS plus IFN-*γ*-induced RAW264.7 cells. The intracellular ROS levels are expressed as a percentage of the control. (c) The effects of GIE on antioxidant mRNA expression in LPS plus IFN-*γ*-induced RAW264.7 cells. (d) GIE suppressed NO production in LPS plus IFN-*γ*-induced RAW264.7 cells. Nitrite concentration was determined from a sodium nitrite standard curve, and the results are expressed as a concentration (*μ*M) of nitrite in a culture medium. (e) The effects of GIE on COX-2 and iNOS mRNA expression in LPS plus IFN-*γ*-induced RAW264.7 cells. The data represent the mean ± S.D. of two independent experiments. Cells were pretreated with GIE, NAC, or DEX for 3 h and then coincubated with LPS plus IFN-*γ* for 24 h. UN: uninduced cells; IN: untreated LPS plus IFN-*γ*-induced cells; NAC: cells were pretreated with NAC at 3 mM; DEX: cells were pretreated with DEX at 1 *μ*M; GIE (50, 100, 200, and 300): cells were pretreated with GIE at concentrations range of 50, 100, 200, and 300 *μ*g/mL, respectively. One-way ANOVA performed the comparison, and Tukey was used as a *post hoc* test. The degree of significance was denoted with different letters for the comparison between sample groups. *p* < 0.05 was considered as statistically significant.

The cytotoxicity of GIE was evaluated by MTT assay. The RAW264.7 cells were treated with various concentrations of the GIE, ranging from 100 to 500 *μ*g/mL for 24 h. As shown in Figure 1(a), the results demonstrated that GIE at the concentration ranges of 100-400 *μ*g/mL did not show a toxic effect on RAW264.7 cells (*p* > 0.05). At the highest tested concentration (500 *μ*g/mL), the number of living RAW264.7 cells decreased by up to 71.50% compared to the control group. Based on these results, the nontoxic concentration ranges of GIE (50, 100, 200, and 300 *μ*g/mL) were selected for treating cells in further investigation.

### 3.4. GIE Attenuated the Intracellular ROS Generation and Increased SOD2 mRNA Expression in LPS plus IFN-*γ*-Induced RAW264.7 Cells

ROS generated by inflammatory cells also stimulates pathways that lead to the amplification of inflammation. ROS-induced kinase activation leads to the activation of transcription factors, which triggers the generation of proinflammatory cytokines and chemokine. Therefore, it is possible that the inflammation would be controlled by suppressing intracellular ROS production. Antioxidants are proved that help to protect cells and tissue from damage caused by free radical molecules [43]. According to FRAP and DPPH results, the possibility of GIE to scavenge intracellular ROS formation was further evaluated using the cell-base assay, DCFH-DA model. LPS and IFN-*γ* were chosen as an inflammatory inducing molecule, which can trigger the generation of a series of inflammatory mediators and reactive oxygen. NAC, as a nutritional supplement, was applied as the positive antioxidant control. The cells were pretreated with various concentrations of GIE (50, 100, 200, and 300 *μ*g/mL) for 3 h and then coincubated with LPS plus IFN-*γ* for another 24 h. As shown in Figure 1(b), while LPS plus IFN-*γ* increased ROS formation in RAW264.7 cells (by 1.76-fold compared to uninduced cells), pretreated cells with GIE significantly reduced ROS generation in a concentration-dependent manner. Surprisingly, the highest concentration of GIE showed the efficacy of decreasing the intracellular ROS level to 56.72 ± 3.62%, which was a similar level of the basal intracellular ROS production (56.56 ± 3.18%) in uninduced cells (UN). As expected, the positive antioxidant compound, NAC, possessed a potent free radical scavenging activity by decreasing the intracellular ROS level to 44.07 ± 3.82% compared to untreated LPS plus IFN-*γ*-induced cells. These results lead us to believe that GIE could inhibit ROS production by scavenging free radicals in cells, which is confirmed by the results of the FRAP and DPPH assay (Table 3). Our study was in agreement with other studies, which reported that *G. inodorum* displayed the antioxidant activity measuring by various *in vitro* antioxidant assays [13, 44]. However, this is the first report of the study, proving the antioxidant effect of this plant in the cell-based assay. Next, the inhibitory effect of GIE on oxidative stress was investigated by measuring antioxidant enzyme mRNA expression in RAW264.7 cells induced by LPS plus IFN-*γ*. Following stimulation of the cells by LPS plus IFN-*γ*, SOD2 mRNA expression exhibited a slight increase, along with a significant decrease in GSTP1, NQO1, GCLC, and GCLM mRNA expression (Figure 1(c)). Treatment by 300 *μ*g/mL GIE achieved a statistically significant increase in SOD2 mRNA expression in LPS plus IFN-*γ*-induced cells (*p* < 0.05). However, with concentrations of 300 *μ*g/mL, there was no statistically significant difference in the mRNA expression of GSTP1, NQO1, GCLC, and GCLM compared to LPS plus IFN-*γ*-induced cells (IN) (*p* > 0.05). These results provide evidence that GIE inhibited ROS production by upregulating the expression of SOD2 mRNA levels in LPS plus IFN-*γ*-induced RAW264.7 cells.

ROS is considered to be a causal factor in inflammatory responses. Higher levels of ROS can cause toxicity or act as signaling molecules. The cellular levels of ROS are controlled by low molecular mass antioxidants and antioxidant enzymes. SOD2 is one of the primary cellular antioxidant enzymes, which catalyze the dismutation of superoxide anion (O_2_^−^) to oxygen and hydrogen peroxide (H_2_O_2_) [45]. SOD2 was actively expressed via the NF-*κ*B pathway during the progression of inflammatory conditions. The intracellular SOD2 has a protective role by suppressing the nucleotide-binding oligomerization domain, leucine-rich repeat, and pyrin domain-containing (NLRP) inflammasome-caspase-1-IL-1*β* axis under inflammatory conditions [46]. Superoxide dismutase (SOD) also acts as an anti-inflammatory due to its inhibitory effects on the release of lipid peroxidation-derived prostaglandins, thromboxane, and leukotrienes [47]. Therefore, GIE elevated the levels of SOD2 can be an effective therapeutic strategy in oxidative stress and inflammation.

### 3.5. GIE Suppressed the Production of NO and Attenuated iNOS and COX-2 mRNA Expression in LPS plus IFN-*γ*-Induced RAW264.7 Cells

NO is a reactive nitrogen species (RNS), which also plays essential biology roles, similar to ROS. NO is synthesized by activating macrophages involved in an immune and inflammatory response. Inhibition of NO production was usually used as the necessary pharmacological treatment of inflammation-related diseases. Therefore, in this study, we investigated whether GIE could modulate NO production in LPS plus IFN-*γ*-induced RAW264.7 cells and measured for NO production using the Griess assay. The anti-inflammatory agent, dexamethasone (DEX), was used as the reference drug. As shown in Figure 1(d), the results showed that LPS plus IFN-*γ* induced significantly increased NO production (*p* < 0.05), reaching 45.68 ± 1.26 *μ*M in untreated LPS plus IFN-*γ*-induced cells (IN). GIE reduced NO production in a dose-dependent manner. Moreover, the extract at concentrations of 200 and 300 *μ*g/mL significantly suppressed NO production compared to untreated LPS plus IFN-*γ*-induced cells (IN) (*p* < 0.05). The highest concentration of GIE at 300 *μ*g/mL exhibited the NO suppression (12.62% of NO inhibition), approximately the same efficiency as the reference drug, DEX (15.80% of NO inhibition).

In the inflammatory response, NO and PGE_2_ are synthesized by iNOS and COX-2, respectively [48]. To confirm if the suppression of NO production by GIE was related to change in iNOS as well as COX-2 mRNA levels, qRT-PCR was performed.  Figure 1(e) showed that iNOS and COX-2 mRNA expression was increased in LPS plus IFN-*γ*-induced cells (IN). At the same time, pretreated GIE significantly decreased the expression of iNOS and COX-2 mRNA levels in a concentration-dependent manner (*p* < 0.05). The present study indicates that the suppressive effect of GIE on NO production is mediated through the inhibition of iNOS mRNA expression. Therefore, iNOS reduction leads to the lower of PGE_2_ and COX-2 expression in activated macrophages with LPS. There is no doubt that LPS and IFN-*γ* also efficiently enhance COX-2 expression in RAW264.7 cells [49]. Our results demonstrate that GIE can exhibit anti-inflammatory activity by attenuating COX-2 mRNA expression. Thus, GIE might play essential roles in ameliorating inflammation by suppressing NO production and downregulation of iNOS and COX-2 mRNA expression.

### 3.6. GIE Suppressed Proinflammatory Cytokines (IL-6 and TNF-*α*), Proinflammatory mRNA Expression, and Slightly Increased Anti-inflammatory Cytokines (IL-10) in LPS plus IFN-*γ*-Induced RAW264.7 Cells

**Figure 2:**
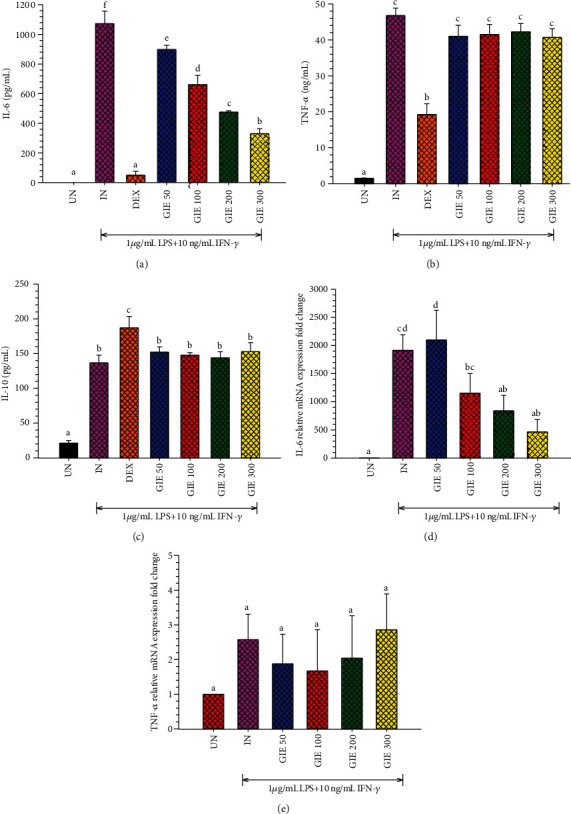
GIE suppressed the secretion of (a) proinflammatory cytokines IL-6, (b) TNF-*α*, and slightly increased (c) anti-inflammatory cytokines IL-10 secretion in LPS plus IFN-*γ*-induced RAW264.7 cells. The effects of GIE on (d) IL-6 and (e) TNF-*α* mRNA expression. Cells were pretreated with GIE or DEX for 3 h and then coincubated with LPS plus IFN-*γ* for 24 h. UN: uninduced cells; IN: untreated LPS plus IFN-*γ*-induced cells; DEX: cells were pretreated with DEX at 1 *μ*M; GIE (50, 100, 200, and 300): cells were pretreated with GIE at concentrations range of 50, 100, 200, and 300 *μ*g/mL, respectively. The data represent the mean ± S.D. of two independent experiments. One-way ANOVA performed the comparison, and Tukey was used as a *post hoc* test. The degree of significance was denoted with different letters for the comparison between sample groups. *p* < 0.05 was considered as statistically significant.

Inflammation is mediated by cytokines released from immune cells in response to pathogens' molecular components, such as the LPS of gram-negative bacteria. TNF-*α* in inflammatory processes is an essential proinflammatory mediator, leading to other inflammatory molecular expressions as IL-6 and COX-2. However, IL-10 is a key anti-inflammatory cytokine with immunomodulatory effects, causing the inhibition of another proinflammatory cytokine such as TNF-*α* [50]. In order to confirm the anti-inflammatory activity of the GIE, the effects of the extract on proinflammatory cytokine (IL-6 and TNF-*α*) and anti-inflammatory cytokine (IL-10) production were evaluated in LPS plus IFN-*γ*-induced RAW264.7 cells by using ELISA. The results indicated that GIE at all tested-concentration significantly suppressed LPS plus IFN-*γ*-induced IL-6 production (*p* < 0.05; Figure 2(a)) and slightly decreased TNF-*α* production compared to untreated LPS plus IFN-*γ*-induced cells (IN) (Figure 2(b)). As expected, DEX (a reference drug) could also inhibit LPS plus IFN-*γ*-induced IL-6 and TNF-*α* production by 95.38% and 58.88%, respectively. Surprisingly, LPS plus IFN-*γ* induced the secretion of IL-10 about 6.5-fold compared to uninduced cells. DEX significantly increased the secretion of IL-10 level by almost 36.78% compared to untreated LPS plus IFN-*γ*-induced cells (*p* < 0.05; Figure 2(c)). While pretreated-GIE only showed a slight increase in IL-10 levels (around 5-10%) but no significant difference compared to untreated LPS plus IFN-*γ*-induced cells (*p* > 0.05). qRT-PCR was performed to investigate whether suppression of TNF-*α* and IL-6 production by GIE was related to a change in mRNA levels for both proinflammatory cytokines. Increasing concentrations of GIE produced a decrease in the order of IL-6 mRNA levels in LPS plus IFN-*γ*-induced cells (Figure 2(d)). However, GIE slightly decreased TNF-*α* mRNA levels compared to LPS plus IFN-*γ*-induced cells (Figure 2(e)). The profile of IL-6 and TNF-*α* suppression by GIE suggests that GIE acts more potent on IL-6 than TNF-*α*.

Base on GC-MS analysis, the volatile oil compounds presenting in GIE, including phytol, squalene, *γ*-tocopherol, dl-*α*-tocopherol, and stigmasterol, have been reported to have anti-inflammatory and antioxidant activities. Therefore, the anti-inflammatory effects of the GIE could simply rely on volatile oil components that may exert synergistic effects. This is the first study concerning the inhibitory activity against proinflammatory cytokines (IL-6 and TNF-*α*) in LPS plus IFN-*γ*-induced RAW264.7 cells. The secretion of proinflammatory cytokines, TNF-*α* and IL-6, is an important factor in upregulating the inflammatory process. The high levels of TNF-*α* and IL-6 play a critical role in acute and chronic inflammatory diseases; both cytokines are prime targets for intervention by anti-inflammatory therapeutic agents. Therefore, the development of anti-inflammatory substances, which can modulate proinflammatory mediators' production, is an efficient way to manage inflammatory conditions. These results provide evidence that GIE could be a source of anti-inflammatory agents, which may have a beneficial effect on treating inflammation-associated diseases.

### 3.7. Effects of GIE on the Morphology of LPS plus IFN-*γ*-Induced RAW264.7 Cells

**Figure 3:**
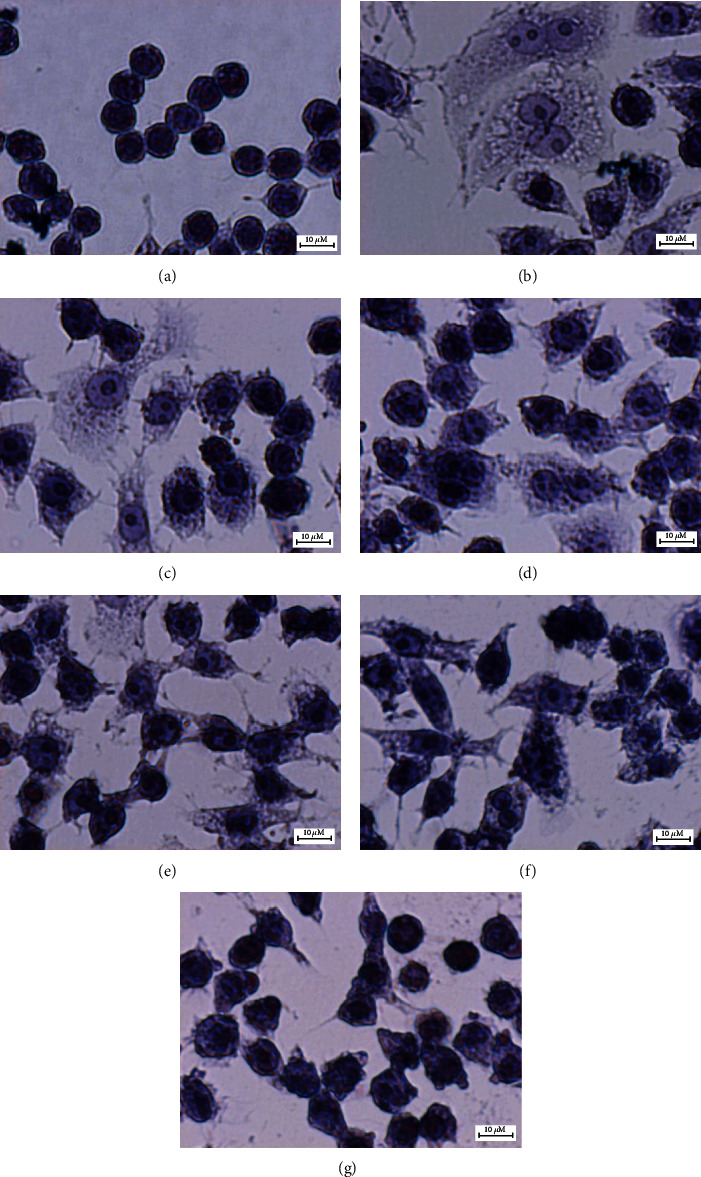
Effects of GIE on the morphology of LPS plus IFN-*γ*-induced RAW264.7 cells. Cells were stained with hematoxylin staining: (a) uninduced RAW264.7 cells, (b) untreated LPS plus IFN-*γ*-induced cells, (c) cells were pretreated with DEX at 1 *μ*M, (d), (e), (f), and (g) cells were pretreated with GIE at concentrations range of 50, 100, 200, and 300 *μ*g/mL, respectively (original magnification at ×600, scale bar; 10 *μ*m).

The morphological alteration in LPS plus IFN-*γ*-induced RAW264.7 cells in the presence or absence of GIE were also observed (as seen in Figures 3(a)–3(g)). The results demonstrated that LPS and IFN-*γ* caused cell morphology to change into a pseudopodia formation, spreading, and pancake-like shape within 24 h of stimulation (Figure 3(b)). The phenotypic polarization of macrophages allows the macrophage to engulf lipids, dead cells, other substances perceived as danger signals and secrete a large number of inflammatory molecules [51, 52]. In contrast, uninduced cells (UN) displayed a circular shape, a common form of RAW264.7 cells (Figure 3(a)). The pretreatment with GIE decreased the degree of cell spreading and pseudopodia formation, which was obviously observed in cells after pretreated with GIE 300 *μ*g/mL (Figure 3(g)). Our results are in substantial agreement with a previous study of Kang et al. that the cotreatment of LPS with the crude methanol extract of *Citrus aurantium* L. reduces the level of cell spreading and pseudopodia formation by suppressing cell differentiation [53].

### 3.8. Effects of GIE on NF-*κ*B p65 Nuclear Translocation and p-NF*κ*B p65 (27.Ser 536) Protein Expression in LPS plus IFN-*γ*-Induced RAW264.7 Cells

**Figure 4:**
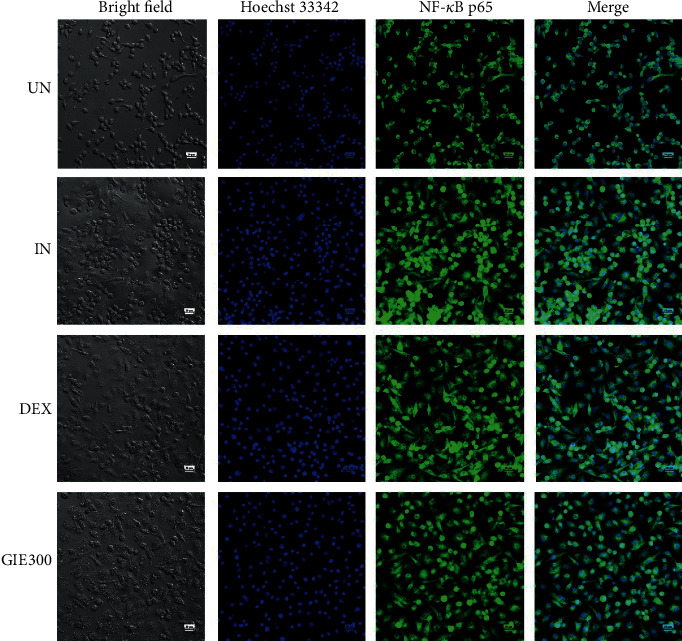
Effects of GIE on the nuclear translocation of NF-*κ*B p65 in LPS plus IFN-*γ*-induced RAW264.7 cells at 24 h. Cells were pretreated with GIE or DEX for 3 h and then coincubated with LPS plus IFN-*γ* for 24 h. The nuclear translocation of NF-*κ*B p65 was detected using an immunofluorescence assay and visualized under confocal microscopy. The figure represents the cell morphology (bright field), the nuclear translocation of NF-*κ*B p65 (green fluorescence), nucleus (blue fluorescence), and costaining (overlay green and blue fluorescence). Scale bar, 20 *μ*m. UN: uninduced cells; IN: untreated LPS plus IFN-*γ*-induced cells; DEX: cells were pretreated with DEX at 1 *μ*M; GIE300: cells were pretreated with GIE 300 *μ*g/mL.

Given that LPS plus IFN-*γ*-induced inflammation is through Toll-like receptor (TLR) signaling, NF-*κ*B is activated and translocate into the nucleus to regulate the induced transcription of proinflammatory genes [54]. As a marker of NF-*κ*B activation, the nuclear translocation of the NF-*κ*B p65 was visualized in LPS plus IFN-*γ*-induced RAW264.7 cells by immunofluorescence confocal microscopy. As shown in Figure 4, LPS plus IFN-*γ*-induced RAW264.7 cells (IN) showed marked NF-*κ*B p65 staining in the nuclei, while uninduced cells (UN) showed weaker nuclear NF-*κ*B expression but stronger staining in the cytoplasm. GIE treatment (GIE300) attenuated LPS plus IFN-*γ*-induced nuclear translocation of NF-*κ*B p65. Based on these findings, GIE can decrease the nuclear translocation of NF-*κ*B, thus further inhibiting the expression of target inflammatory genes.

**Figure 5:**
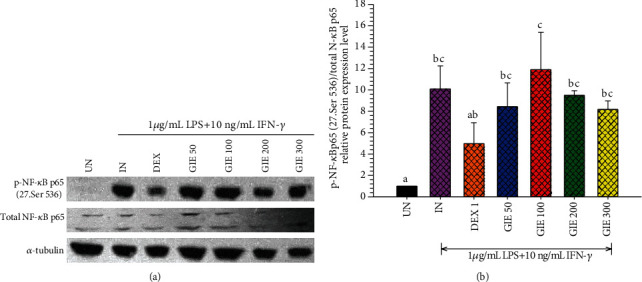
Effects of GIE on phosphorylation of NF-*κ*B p65 induced by LPS plus IFN-*γ* in RAW264.7 cells. Cells were pretreated with GIE or DEX for 3 h and then coincubated with LPS plus IFN-*γ* for 24 h. The protein expression was analyzed by Western blotting. (a) The cellular proteins were used to detect the phosphorylated NF-*κ*B p65 and total form of NF-*κ*B with *α*-tubulin as a housekeeping control protein. (b) Mean densitometric values are expressed as bar charts. The data represent the mean ± S.D. of three independent experiments. One-way ANOVA performed the comparison, and Tukey was used as a *post hoc* test. The degree of significance was denoted with different letters for the comparison between sample groups. *p* < 0.05 was considered as statistically significant. UN: uninduced cells; IN: untreated LPS plus IFN-*γ*-induced cells; DEX: cells were pretreated with DEX at 1 *μ*M; GIE300: cells were pretreated with GIE 300 *μ*g/mL.

To further investigate whether GIE can regulate NF-*κ*B signaling in LPS plus IFN-*γ*-induced RAW264.7 cells, the phosphorylation of NF-*κ*B p65 was detected by Western blotting. Stimulation of the uninduced cells by LPS plus IFN-*γ* (IN) exhibited significantly higher phosphorylation of NF-*κ*B p65 than uninduced cells (UN) (*p* < 0.05; Figures 5(a) and 5(b)). Pretreatment with 1 *μ*M DEX or GIE showed a trend to reduce the phosphorylation of NF-*κ*B p65 proteins, but no significant difference compared to LPS plus IFN-*γ*-induced cells (IN) (*p* > 0.05). This result suggests that GIE exhibits a slight inhibitory effect on the phosphorylation of NF-*κ*B p65 to exert its anti-inflammatory effects.

In oxidative stress and anti-inflammation, enhancement of antioxidant gene expression plays an essential role in cell protection. It has been reported that superoxide dismutase (SOD) can modulate ROS-dependent signaling pathways during inflammatory responses [55]. The activation of Nrf2 antioxidant pathway prevents LPS-induced transcriptional upregulation of proinflammatory cytokines [56]. This research found that GIE increased the antioxidant gene expression, SOD2, and reduced NO and ROS production. Therefore, it is possible that the antioxidant properties of GIE could modulate the inflammation process caused by regulating the ROS levels.

### 3.9. Vitamin E Exhibited Anti-inflammatory Activities by Suppressing iNOS, COX-2, and IL-6 mRNA Expression in LPS plus IFN-*γ*-Induced RAW264.7 Cells

**Figure 6:**
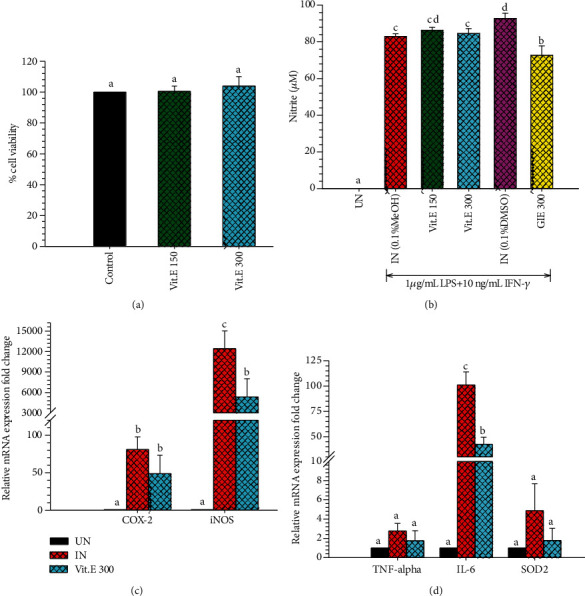
(a) Cytotoxic effects of Vit.E on RAW264.7 cells for 24 h. MTT assay was used to determine cell viability. Values are expressed as a percentage of the control. (b) The effect of Vit.E compared to GIE on NO production in LPS plus IFN-*γ*-induced RAW264.7 cells. Nitrite concentration was determined from a sodium nitrite standard curve, and the results are expressed as a concentration (*μ*M) of nitrite in a culture medium. The data represent the mean ± S.D. of three independent experiments. (c) The effects of Vit.E on COX-2 and iNOS mRNA expression in LPS plus IFN-*γ*-induced RAW264.7 cells. (d) The effects of Vit.E on TNF-*α*, IL-6, and SOD2 mRNA expression in LPS plus IFN-*γ*-induced RAW264.7 cells. The data represent the mean ± S.D. of two independent experiments. Cells were pretreated with GIE or DEX for 3 h and then coincubated with LPS plus IFN-*γ* for 24 h. UN: uninduced cells; IN: untreated LPS plus IFN-*γ*-induced cells; DEX: cells were pretreated with DEX at 1 *μ*M; GIE 300: cells were pretreated with GIE 300 *μ*g/mL; Vit.E (150 and 300): cells were pretreated with Vit.E 150 and 300 *μ*g/mL, respectively. One-way ANOVA performed the comparison, and Tukey was used as a *post hoc* test. The degree of significance was denoted with different letters for the comparison between sample groups. *p* < 0.05 was considered as statistically significant.

GIE has been reported to have antioxidant and anti-inflammatory activities in LPS plus IFN-*γ*-induced RAW264.7 cells in this research. Based on GC-MS analysis, we found that GIE consisted of many volatile oil compounds, including phytol, squalene, *γ*-tocopherol, dl-*α*-tocopherol, and stigmasterol. The antioxidant and anti-inflammatory activities of these active constituents have been demonstrated in several studies. Therefore, it is essential to confirm whether the active ingredients contributed to the antioxidant and anti-inflammatory abilities of GIE. The dl-alpha-tocopherol or Vit.E was *chosen* to elucidate the anti-inflammation and antioxidative activities in LPS plus IFN-*γ*-induced RAW264.7 cells. The cytotoxicity of Vit.E was evaluated. The results exhibited that Vit.E at 150 and 300 *μ*g/mL did not reduce the cell viability compared to RAW264.7 cells (Figure 6(a)). Then, the noncytotoxic concentration of Vit.E at 300 *μ*g/mL was chosen to perform subsequent experiments. Vit.E did not suppress NO production induced by LPS plus IFN-*γ* in RAW264.7 cells (Figure 6(b)). The results indicated that Vit.E showed a significantly suppressed iNOS and IL-6 mRNA expression lower than untreated LPS plus IFN-*γ*-induced cells (IN) (*p* < 0.05; Figures 6(c) and 6(d)), whereas COX-2, TNF-*α*, and SOD2 were lower than IN but no significant difference (*p* > 0.05; Figures 6(c) and 6(d)). Therefore, these results imply that Vit.E exhibits the inhibitory effect on the inflammatory gene expression, which is related to the anti-inflammatory effects of GIE. As mentioned above, GIE consists of many volatile oils; therefore, the antioxidant and anti-inflammatory activities of it may cause by other chemical compositions found in this plant. Moreover, phenolic, flavonoids, terpenoids, and glycoside in GIE were reported in previous studies [14, 15]. The presence of flavonoids had been reported to be associated with the antioxidant and anti-inflammatory activities of several plant extracts [25, 57, 58]. Thus, it is possible that phenolic and flavonoid compounds in GIE could provide substantial antioxidant and anti-inflammatory activities. In order to compare the inhibitory effect of Vit.E and GIE, GIE at 300 *μ*g/mL contains Vit.E approximately 6.99 *μ*g/mL. The results indicated that GIE at 300 *μ*g/mL had inhibitory effect on COX-2 (73.48% inhibition, Figure 1(e)), iNOS (64.58% inhibition, Figure 1(e)), and IL-6 (75.72% inhibition, Figure 2(d)) mRNA levels higher than Vit.E alone at 300 *μ*g/mL, which inhibited COX-2 (40.74% inhibition, Figure 6(c)), iNOS (56.86% inhibition, Figure 6(c)), and IL-6 (58.42% inhibition, Figure 6(d)). These results imply that the anti-inflammatory effect of GIE on these cells may have synergistic activities that cause by the combination of compounds presenting in GIE.

### 3.10. SR-FTIR Detected Biomolecule Alterations in RAW264.7 Cells

**Figure 7:**
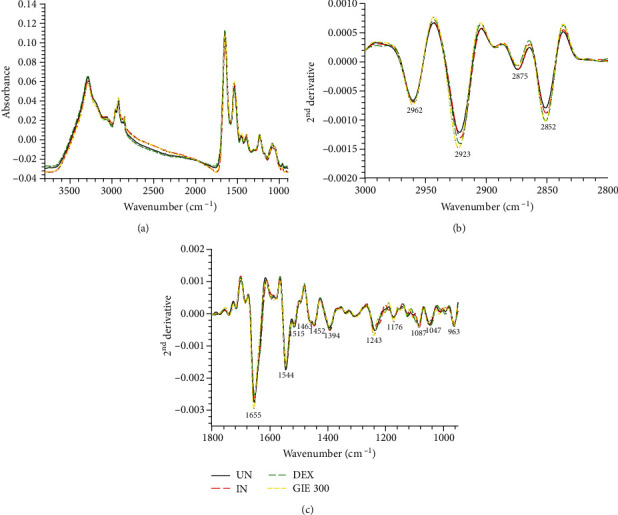
FTIR spectra obtained using SR-FTIR and processed with a second derivative. (a) Average original FTIR spectra (3800-900 cm^−1^), (b) average the second derivative spectra of lipid regions (3000-2800 cm^−1^), and (c) average the 2^nd^ derivative spectra of protein and nucleic acid and other carbohydrate regions (1800-950 cm^−1^). The data obtained from uninduced cells (UN, *n* = 70), untreated LPS plus IFN-*γ*-induced cells (IN, *n* = 70), 1 *μ*M of DEX-treated cells (DEX, *n* = 70), and 300 *μ*g/mL of GIE-treated LPS plus IFN-*γ*-induced cells (GIE300, *n* = 70).

**Figure 8:**
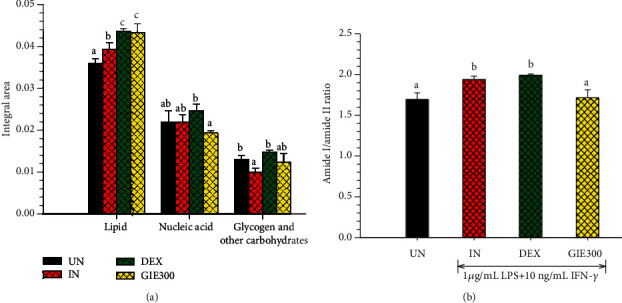
Bar graph shows (a) integrated areas of remarkable lipid, nucleic acid, glycogen, and other carbohydrate regions and (b) the amide I/amide II ratio of 2^nd^ derivative spectra. Data are represented as means ± S.D. for three replicates. The data obtained from uninduced cells (UN, *n* = 70), untreated LPS plus IFN-*γ*-induced cells (IN, *n* = 70), 1 *μ*M of DEX-treated cells (DEX, *n* = 70), and 300 *μ*g/mL of GIE-treated LPS plus IFN-*γ*-induced cells (GIE300, *n* = 70). One-way ANOVA performed the comparison, and Tukey was used as a *post hoc* test. The degree of significance was denoted with different letters for the comparison between sample groups. *p* < 0.05 was considered as statistically significant.

SR-FTIR was applied to detect the effects of GIE on biomolecule alterations such as lipids, proteins, nucleic acids, and carbohydrates based on their specific vibrational fingerprints. The detailed spectral band assignments of samples are given in Table 4. As shown in Figure 7(a), the representative FTIR spectra acquired in the 3800-900 cm^−1^ from four individuals of sample groups, including uninduced RAW264.7 cells, untreated LPS plus IFN-*γ*-induced RAW264.7 cells, 1 *μ*M of DEX-, and 300 *μ*g/mL GIE-treated LPS plus IFN-*γ*-induced RAW264.7 cells. The second derivative analysis in spectral regions ranges from 3,000 to 2800 cm^−1^ for lipid regions and 1800 to 950 cm^−1^ for protein regions, nucleic acid, and other carbohydrate regions (as shown in Figures 7(b) and 7(c)) respectively. The 2^nd^ spectral revealed the prominent differences between the average spectra belonging to the different groups. The spectral differences were clearly observed mainly in lipid regions centered at 2962 cm^−1^, 2923 cm^−1^, and 2852 cm^−1^, protein regions centered at 1655 cm^−1^ and 1544 cm^−1^, nucleic acid and other carbohydrates regions centered at 1243 cm^−1^, 1176 cm^−1^, 1087 cm^−1^, and 1047 cm^−1^. The integral area of 2^nd^ derivative FTIR spectral regions in each region was calculated to analyze the differences between lipid regions (2972-2951 cm^−1^, 2934-2910 cm^−1^, 2878-2870 cm^−1^, and 2856-2843 cm^−1^), nucleic acids (1257-1204 cm^−1^ and 1125-1074 cm^−1^), glycogen, and other carbohydrates (1181-1164 cm^−1^ and 1063-1032 cm^−1^) as shown in Figure 8(a). The level of lipid content was significantly increased in all test groups compared to the uninduced group (*p* < 0.05), whereas the highest level of lipid was found in DEX- and GIE-treated groups. This increase in lipid content might be due to the changing of the cell membrane and lipid accumulation in RAW264.7 cells. According to the results of cell morphological changes stained by hematoxylin staining, the phenotypic changes of RAW264.7 cells are induced by LPS and IFN-*γ* (Figures 3(b)–3(g)). The increasing lipid content may be related to the inflammatory phenotype of macrophages in which they are able to engulf lipids, dead cells, and other substances perceived as danger signals via phagocytosis [52]. All ingested lipids are then processed by acid lipases within the lysosomes, leading to free fatty acids and cholesterol generation [59]. Nevertheless, our data indicated that nucleic acid content seemed to increase in the DEX-treated cells and decrease in GIE-treated cells. However, the differences were not statistically significant from both uninduced cells and untreated LPS plus IFN-*γ*-induced cells (*p* > 0.05). According to these findings, the present data showed that the selected GIE concentration did not affect the cells' nucleic acids, consistent with the cytotoxic effect of GIE obtained from MTT assay. These results are confirmed by the study of Machana et al. [60], which reported the DNA content in apoptotic cell death, by contrast, to increase during necrotic cell death. As shown in Figure 8(a), the glycogen and other carbohydrate content of the untreated LPS plus IFN-*γ*-induced cells were significantly decreased compared to the uninduced cells (*p* < 0.05). Upon DEX and GIE treatment, the glycogen and other carbohydrate contents were increased compared to the untreated LPS plus IFN-*γ*-induced cells. The band area ratio of amide I (1670-1627 cm^−1^)/amide II regions (1558-1505 cm^−1^) was calculated to obtain information about changes in protein composition and structure (Figure 8(b)). There was a significant increase in the ratio of amide I to amide II bands in untreated LPS plus IFN-*γ*-induced RAW264.7 cells compared to the uninduced RAW264.7 cells (*p* < 0.05), revealing an alteration in the structures of proteins. In comparison, the GIE treating group showed a significant decrease in amide I/amide II ratio compared to untreated LPS plus IFN-*γ*-induced RAW264.7 cells (*p* < 0.05). According to amide I and amide II profiles depending on the protein structural composition, the changing of amide I/amide II ratio suggests that there are some alterations in protein structure and conformation in LPS plus IFN-*γ*-induced RAW264.7 cells [18].

**Figure 9:**
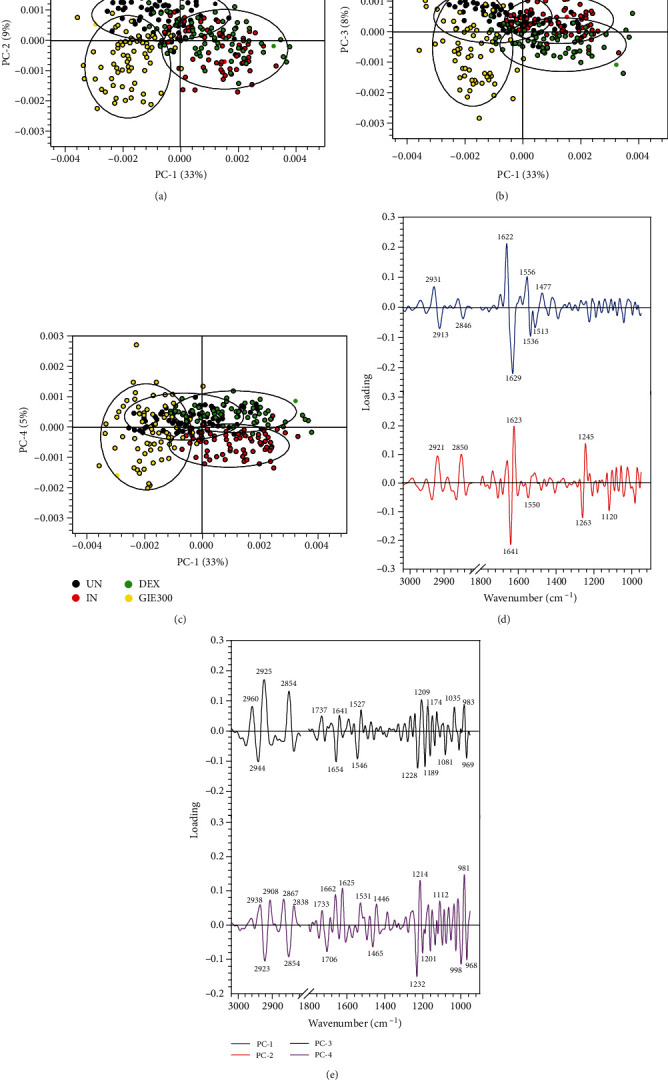
PCA analysis of the FTIR spectra of uninduced cells (UN, *n* = 70), untreated LPS plus IFN-*γ*-induced cells (IN, *n* = 70), 1 *μ*M of DEX-treated cells (DEX, *n* = 70), and 300 *μ*g/mL of GIE-treated LPS plus IFN-*γ*-induced cells (GIE300, *n* = 70) in the spectral range of 3000-2800 cm^−1^ and 1800-950 cm^−1^ regions. PCA 2D score plot of (a) PC1 versus PC2, (b) PC1 versus PC3, and (c) PC1 versus PC4. PCA loading plot of (d) PC1 and PC2 and (e) PC3 and PC4.

Based on the spectral differences, the PCA analysis was applied to confirm any possible discrepancies between four sample groups in spectral ranges of 3000-2800 cm^−1^ and 1800-950 cm^−1^. Four clusters of spectra were distinctly visualized in two-dimensional score plots, including PC1 vs. PC2, PC1 vs. PC3, and PC1 vs. PC4 score plot as shown in Figures 9(a)–9(c), respectively. PCA loading plots were used to indicate the contribution of variables between sample groups (as shown in Figures 9(d) and 9(e)). The PCA analysis obviously illustrated that the clusters of untreated LPS plus IFN-*γ*-induced RAW264.7 cells (IN) and DEX-treated cells (DEX) were separated from the cluster of GIE-treated cells (GIE300) along PC1 (33%). They were also separated from the group of uninduced RAW264.7 cells (UN) along with the PC2 score (9%) as shown in Figure 9(a). The amide I band from protein at 1629 cm^−1^ and 1623 cm^−1^ (assigned to the *β*-sheet structure) were heavily loaded for negative PC1 and positive PC2 loading, respectively (Figure 9(d)), which were responsible for discriminating the untreated LPS plus IFN-*γ*-induced RAW264.7 cells and DEX-treated cells, respectively. These data indicated that the amide I protein of the untreated LPS plus IFN-*γ*-induced RAW264.7 cells and DEX-treated cells were higher than the uninduced and GIE-treated cells. The cluster of DEX-treated cells was discriminated from uninduced and untreated LPS plus IFN-*γ*-induced RAW264.7 cells along PC3 (8%) (Figure 9(b)) in correlation with the positive loading centered at 2960 cm^−1^, 2925 cm^−1^, and 2854 cm^−1^, assigned to the C-H stretching vibration of lipid in the cells (Figure 9(e)). Moreover, the cluster of uninduced and DEX-treated cells could be distinguished from untreated LPS plus IFN-*γ*-induced RAW264.7 cells by their having positive PC4 scores (5%) (Figure 9(c)), which were associated with the negative loading plot centered at 1232 cm^−1^, 1201 cm^−1^, and 998 cm^−1^, attributing to C–C from nucleic acid and C–O from a glycoprotein and other carbohydrates. This data displayed that the glycogen and other carbohydrates in uninduced and DEX-treated cells were higher than untreated LPS plus IFN-*γ*-induced RAW264.7 cells. These results provide evidence that PCA analysis has corresponded to the integrated peak areas obtained from 2^nd^ derivative spectra (Figures 8(a) and 8(b)).

## 4. Conclusions

To our knowledge, the present study is the first report of the antioxidant and anti-inflammatory effects of GIE in LPS plus IFN-*γ*-induced RAW264.7 cells. GIE exerted an antioxidative effect based on its ROS scavenging properties and elevating the antioxidant gene expressions. GIE possesses anti-inflammatory effects by suppressing both proinflammatory mediators and gene expression in LPS plus IFN-*γ*-induced RAW264.7 cells. The data from SR-FTIR spectroscopy exhibited that LPS plus IFN-*γ* could affect the biochemical profile of RAW264.7 cells. SR-FTIR analysis was able to evaluate the effect of GIE on the changing of macromolecules, including lipid, protein structure, nucleic acid, glycogen, and other carbohydrates. These findings provide evidence that GIE has significant antioxidant and anti-inflammatory properties and could serve as a potent compound of those activities that warrant further research and development.

## Figures and Tables

**Table 1:** Oligomeric nucleotide primer sequence of qRT-PCR.

Gene	Forward primer (5′-3′)	Reverse primer (5′-3′)
iNOS	CAGCACAGGAAATGTTTCAGC	TAGCCAGCGTACCGGATGA
COX-2	TTTGGTCTGGTGCCTGGTC	CTGCTGGTTTGGAATAGTTGCTC
IL-6	CTGCAAGAGACTTCCATCCAG	AGTGGTATAGACAGGTCTGTTGG
TNF-*α*	CAGGCGGTGCCTATGTCTC	CGATCACCCCGAAGTTCAGTAG
SOD2	TTAACGCGCAGATCATGCA	GGTGGCGTTGAGATTGTTCA
GSTP1	TGGGCATCTGAAGCCTTTTG	GATCTGGTCACCCACGATGAA
NQO1	TTCTGTGGCTTCCAGGTCTT	AGGCTGCTTGGAGCAAAATA
GCLC	GATGATGCCAACGAGTCTGA	GACAGCGGAATGAGGAAGTC
GCLM	CTGACATTGAAGCCCAGGAT	GTTCCAGACAACAGCAGGTC
GAPDH	AACGACCCCTTCATTGAC	TCCACGACATACTCAGCAC

**Table 2:** GC-MS analysis of GIE.

No.	Peak name	Formula	Molecular weight (g/mol)	RT (min)	%Area
1	2,3-Dihydrobenzofuran	C_8_H_8_O	120.15	4.74	3.76
2	2-Methoxy-5-vinylphenol	C_9_H_10_O_2_	150.17	5.89	1.42
3	Methylparaben	C_8_H_8_O_3_	152.15	7.71	11.58
4	Tetradecanoic acid	C_14_H_28_O_2_	228.37	11.09	2.86
5	n-Hexadecanoic acid	C_16_H_32_O_2_	256.43	14.06	16.98
6	Phytol	C_20_H_40_O	128.17	16.73	5.96
7	Linolenic acid	C_18_H_30_O_2_	278.43	17.42	24.91
8	Octadecanoic acid	C_18_H_36_O_2_	284.48	17.70	2.04
9	Glycerol beta-palmitate	C_19_H_38_O_4_	330.50	23.60	5.54
10	Beta-Monolinolein	C_21_H_38_O_4_	354.52	26.36	6.31
11	9,12,15-Octadecatrienal	C_18_H_30_O	262.43	26.51	9.50
12	Squalene	C_30_H_50_	410.72	28.27	0.93
13	*γ*-Tocopherol	C_28_H_48_O_2_	416.68	31.37	1.80
14	dl-*α*-Tocopherol	C_29_H_50_O_2_	430.72	33.04	2.33
15	Stigmasterol	C_29_H_48_O	412.69	35.67	2.50
16	Olean-12-ene-3,28-diol, (3beta)-	C_30_H_50_O_2_	442.73	46.98	1.57

**Table 3:** The FRAP and DPPH scavenging activities of GIE and standard compounds.

Sample	FRAP values		DPPH scavenging activity (IC_50_) *μ*g/mL
	(*μ*g VCEA/mg of dry extract)	(*μ*g TREA/mg of dry extract)	
GIE	24.00 ± 0.69	28.06 ± 0.78	406.59 ± 0.11^c^
Vitamin C	—	—	44.57 ± 0.59^a^
Trolox	—	—	67.19 ± 4.82^b^

Values are mean ± S.D. (*n* = 3) and are representative of three independent experiments with similar results. One-way ANOVA performed the comparison, and Tukey was used as a *post hoc* test. The degree of significance was denoted with different letters for the comparison between sample groups. *p* < 0.05 was considered as statistically significant.

**Table 4:** Band assignments of major absorptions in FTIR spectra in 3000-950 cm^−1^ regions.

Band position of 2^nd^ derivative spectra (cm^−1^)	Assignments
2962	CH_3_ stretching (antisymmetric) due to the methyl terminal of membrane phospholipids
2923	CH_2_ antisymmetric stretching of methylene group of membrane phospholipids
2875	CH_3_ symmetric stretching: lipids and proteins
2852	CH_2_ symmetric stretching: mainly lipids
1655	Amide I: C=O (80%) and C–N (10%) stretching, N–H (10%) bending vibrations: proteins *α*-helix
1544	Amide II: N–H (60%) bending and C–N (40%) stretching vibrations: proteins *α*-helix
1515	CO_2_ antisymmetric stretching
1463	CH_2_ bending vibrations: mainly lipids with little contributions from proteins
1452	CH_2_ bending vibrations: mainly lipids with little contributions from proteins
1394	COO^−^ symmetric stretching: fatty acids and amino acids
1243	PO^2-^asymmetric stretching vibrations: RNA, DNA, and phospholipids
1176	C–O vibrations from glycogen and other carbohydrates
1087	PO^2-^asymmetric stretching vibrations: RNA, DNA, and phospholipids
1047	C–O vibrations from glycogen and other carbohydrates
963	C–C/C–O is stretching of deoxyribose vibration

## Data Availability

The datasets used and analyzed during this study are available from the corresponding author on reasonable request.
